# Epidemiology and Spatiotemporal Patterns of Leprosy Detection in the State of Bahia, Brazilian Northeast Region, 2001–2014

**DOI:** 10.3390/tropicalmed3030079

**Published:** 2018-07-31

**Authors:** Eliana Amorim de Souza, Anderson Fuentes Ferreira, Jorg Heukelbach, Reagan Nzundu Boigny, Carlos Henrique Alencar, Alberto Novaes Ramos

**Affiliations:** 1 Multidisciplinary Institute for Health, Campus Anísio Teixeira, Federal University of Bahia, Vitória da Conquista BA 45.029-094, Brazil; 2 Department of Community Health, School of Medicine, Federal University of Ceará, Fortaleza CE 60430-140, Brazil; anderson_deco.f2@hotmail.com (A.F.F.); heukelbach@web.de (J.H.); reagan.nzundu@gmail.com (R.N.B.); carllosalencar@hotmail.com (C.H.A.); novaes@ufc.br (A.N.R.J.); 3 College of Public Health, Medical and Veterinary Sciences, Division of Tropical Health and Medicine, James Cook University, Townsville 4811, Australia

**Keywords:** leprosy, epidemiology, spatial analysis, prevention and control, Brazil

## Abstract

The detection of leprosy cases is distributed unequally in Brazil, with high-risk clusters mainly in the North and Northeast regions. Knowledge on epidemiology and spatiotemporal patterns of leprosy occurrence and late diagnosis in these areas is critical to improve control measures. We performed a study including all leprosy cases notified in the 417 municipalities of Bahia state, from 2001 to 2014. New case detection (overall and pediatric <15 years) and grade 2 disability (G2D) rates were calculated and stratified according to socio-demographic variables. Spatial analyses were performed to detect high-risk areas for occurrence and late diagnosis. A total of 40,060 new leprosy cases was reported in the period (mean = 2861 cases/year), 3296 (8.2%) in <15-year-olds, and 1921 (4.8%) with G2D. The new case detection rate was 20.41 cases/100,000 inhabitants (95% CI: 19.68–21.17). A higher risk was identified in older age groups (RR = 8.45, 95% CI: 7.08–10.09) and in residents living in the state capital (RR = 5.30, 95% CI: 4.13–6.79), in medium-sized cities (RR = 2.80; 95% CI: 2.50–3.13), and in the west (RR = 6.56, 95% CI: 5.13–8.39) and far south regions of the state (RR = 6.56, 95% CI: 5.13–8.39). A higher risk of G2D was associated with male gender (RR = 2.43, 95% CI: 2.20–2.67), older age (RR = 44.08, 95% CI: 33.21–58.51), Afro-Brazilian ethnicity (RR = 1.59; 95% CI: 1.37–1.85), living in medium-sized cities (RR = 2.60; 95% CI: 2.27–2.96) and residency in the north (RR = 5.02; 95% CI: 3.74–6.73) and far south (RR = 7.46; 95% CI: 5.58–9.98) regions. Heterogeneous space–time patterns of leprosy distribution were identified, indicating high endemicity, recent transmission, and late diagnosis. This heterogeneous distribution of the disease was observed throughout the study period. Leprosy remains a relevant public health problem in Bahia state. The disease has a focal distribution. We reinforce the importance of integrating surveillance, prevention and control actions in regions of higher risk of leprosy detection and late diagnosis, and in the most vulnerable populations.

## 1. Introduction

Leprosy is a Neglected Tropical Disease (NTD), mainly affecting highly vulnerable populations [1,2]. Brazil reported a total of 25,218 leprosy cases in 2016, representing 12% of global cases, and 92% of cases in Latin America [3].

The Brazilian Ministry of Health (MoH) established different control strategies aiming for the reduction of the leprosy burden in the country [4]. The Brazilian guidelines for surveillance, attention and elimination of leprosy define measures focusing on primary health care, within the realm of the nationwide Unified Health System (*Sistema Único de Saúde* [SUS]). Primary health care is defined to be responsible for diagnosis, treatment, prevention of disabilities and surveillance. Cases with complex clinical presentations, such as relapses, children <15 years of age and leprosy reactions should be referred to specialized secondary clinics and tertiary care [4].

The early detection of new cases relies on spontaneous presentation of patients to the health system, active case finding and contact tracing (including clinical examination and Bacillus Calmette-Guérin (BCG) vaccination of contacts) [4]. However, due to operational difficulties within the SUS, there are still shortcomings regarding coverage and quality of control and prevention measures [5,6].

As a result of nationwide implementation of these systematic measures, the detection rate of new cases of leprosy cases increased on a first run, but then decreased steadily during the past years. Despite these achievements, leprosy continues being endemic in Brazil, with an overall detection rate of 12.2 cases per 100,000 inhabitants in 2016 [7].

The main targets of the Global Leprosy Strategy of the World Health Organization (WHO) include G2D and leprosy in <15-year-olds, with reduction of new cases with G2D to <1 case per million population [1]. The occurrence of leprosy in children <15 years indicates active transmission of *Mycobacterium leprae*, while the diagnosis of cases with G2D is considered a strong indicator for late diagnosis [4]. The analysis of both epidemiological indicators reveals operational problems in Brazil’s health services network, indicated by 8.40 G2D cases per million population and 3.63 new cases per 100,000 inhabitants in <15-year-olds [7].

The analysis of leprosy detection patterns in space and time is essential for the description of transmission dynamics, considering the focal epidemiological pattern of leprosy [8]. In Brazil, leprosy shows a heterogeneous spatial distribution, with persistence of areas with different levels of endemicity. Higher detection rates are observed mainly in socioeconomically deprived regions [9]. The North, Northeast and Central West regions present the highest disease burden [7]. In this context, the identification of areas of high endemicity is also an important tool to monitor and evaluate the effectiveness of control measures on a nationwide level [1,4,8,10,11]. However, there are only few systematic studies on spatial patterns of leprosy in Brazil’s Northeast region. 

The present study aims to fill this gap by describing the main epidemiological indicators for leprosy, and by characterizing the spatial and temporal patterns of leprosy detection in the state of Bahia, from 2001 to 2014.

## 2. Methods

### 2.1. Study Area, Population and Design

**Figure 1:**
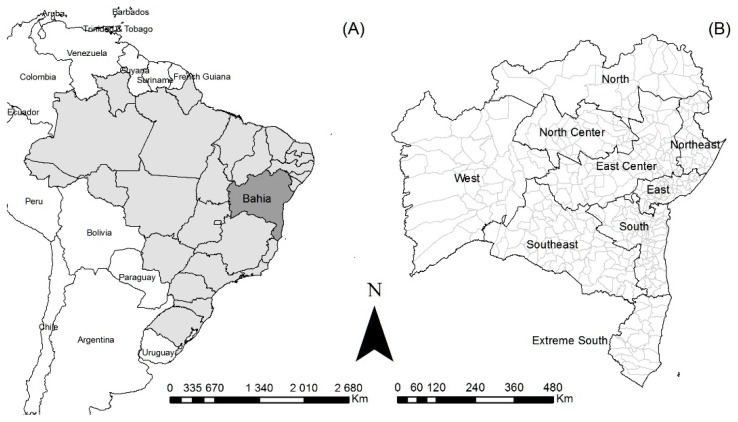
Study area: (**A**) location of the Bahia state; (**B**) Bahia state with its nine regions and 417 municipalities.

Bahia state has a population of about 15 million and is one of the Brazilian states with the highest poverty rates [12,13]. It is the largest state in Brazil’s Northeast (Figure 1). Socio-economic indicators have improved recently (e.g., the Human Development Index [HDI] improved from 0.512 in 2000 to 0.660 in 2010), but social inequality remains at high levels [14]. SUS healthcare coverage, indicated by population coverage of the primary healthcare-based Family Health Strategy (a priority service for development of leprosy control actions) increased from 15.4% in 2001 to 68.9% in 2014 [15].

The study population consisted of all confirmed leprosy cases notified 2001–2014 in inhabitants of Bahia state. We performed an epidemiological analysis of these cases, and spatial analyses in two temporal sections using the 417 municipalities of the state as units [12].

### 2.2. Data Sources and Variables

We used the database of the National Disease Notification System of the MoH (SINAN), formally obtained from the Health Secretariat of Bahia state. SINAN is a standardized software-based system for the compulsory notification of diseases, including leprosy. Information on socio-demographic and clinical data are also available. Only confirmed cases, based on clinical and epidemiological criteria, are reported. For monitoring of cases during treatment, a follow-up report is used by the health services to be completed at the time of discharge [4]. This follow-up report improves quality of SINAN data. In this study, the cases that were defined as ‘diagnostic error’ during follow-up were excluded.

For the calculation of epidemiological indicators, population data were obtained from the Brazilian Institute of Geography and Statistics (IBGE), based on the population censuses of the State (2000 and 2010), and on population estimates for inter-census years (2001–2009; 2011–2014).

### 2.3. Statistical Analyses

#### 2.3.1. Epidemiological Analysis

The following indicators were calculated from secondary SINAN data: (1) annual case detection rate per 100,000 inhabitants, indicating frequency and magnitude; (2) detection rate in <15-year-olds per 100,000 inhabitants, indicating active transmission; and (3) new cases with G2D per 1,000,000 population at the time of diagnosis, indicating under-notification and late diagnosis [4].

Crude rates were calculated per year and the mean rates for the periods 2001–2007 and 2008–2014, as well as the mean for the entire period (2001–2014), smoothing differences over time. The two periods were used considering the first as an initial period of decentralization of control actions for primary health care services and the second, as a phase of consolidation of this process. We used the standardized populations of each period under analysis as the denominator. For all indicators, we calculated their respective binomial 95% confidence intervals (95% CI) [16].

We then stratified the indicators by sociodemographic characteristics: gender, ethnicity (Caucasian, Afro-Brazilian, Asian, Mixed/Pardo-Brazilian, Amerindian), age group (<15, 15–29, 30–39, 40–49, 50–59, 60–69, ≥70 years), residence in the state capital, and size of municipality (small, <100,000 inhabitants; medium size with 100,000–500,000 inhabitants; and large, >500,000 inhabitants). Relative risks (RR) and their 95% CI were calculated to determine the differences between the groups. The statistical significance of differences was evaluated by the chi-square test.

We used the classification of endemicity levels of municipalities, as defined by the Brazilian MoH: the general detection rate was considered hyperendemic when there were >40.00 cases per 100,000 inhabitants; very high >20.00 to 39.99 cases per 100,000 inhabitants; high >10.00 to 19.99 cases per 100,000 inhabitants; medium >2.00 to 9.99 cases per 100,000 inhabitants; and low <2.00 cases per 100,000 inhabitants. Similarly, the detection rate in cases <15 years was classified as low (<0.50/100,000 inhabitants); intermediate (0.5–2.49/100,000 inhabitants); high (2.5–4.99/100,000 inhabitants); very high (5.0–9.99/100,000 inhabitants) and hyperendemic (>10.0/100,000 inhabitants) [4].

#### 2.3.2. Spatial Analyses in the Temporal Sections

Spatial analyses were performed to identify spatial aggregates for the abovementioned leprosy indicators. To reduce random fluctuations (esp. in the case of rare events and small populations) and to minimize the influence of operational factors, the abovementioned indicators were smoothed by applying the local empirical Bayesian method (a procedure for statistical inference in which the prior distribution is estimated from the data). Smoothing is based on information from surrounding municipalities. 

The identification of possible areas of spatial autocorrelation was based on Local Moran’s method (Local Indicators of Spatial Association; LISA), which compares the value of the rate of each municipality and its neighbors, verifying spatial dependence and identifying patterns of spatial autocorrelation. The generated scatter diagram recognizes four situations: municipalities with high or low detection rates, surrounded by municipalities with high or low detection rates (Q1—High-High and Q2—Low-Low) and municipalities with high or low detection rates, surrounded by municipalities with low or high detection rates (Q3—High-Low and Q4—Low-High). For spatial representation, we applied Moran Maps, considering municipalities with a statistically significant difference. The definition of risk areas for the detection of leprosy cases, active transmission and late diagnosis was based on the identification of municipalities with high values of the respective epidemiological indicators.

We also used the Gi* index (‘Gi star’) of Getis-Ord, for the analysis of spatial dependence. The analyses assume that a high value of the Z score and small p value of a parameter indicate spatial agglomeration of high values. A low negative Z score and small p value indicate spatial grouping of low values [17]. These indices identify the presence of aggregates of high-values or low-values within the aggregate of municipalities.

Statistical analyses were performed with Stata version 11.2 (StataCorp LP, College Station, TX, USA). ArcGIS version 9.3 (Environmental Systems Research Institute—ESRI, Redlands, CA, USA) and Terra View version 4.1 (INPE, São José dos Campos, SP, Brazil) were used for spatial analyses, including processing, analysis, presentation of cartographic data and calculations of the indicators of spatial autocorrelation, as well as construction of thematic maps.

The study was conducted in accordance with the Declaration of Helsinki, and the protocol was approved by the Ethical Review Board of the Federal University of Ceará (protocol number: 544,962 28/02/2014).

## 3. Results

### 3.1. Epidemiological Analysis

A total of 40,060 new leprosy cases was notified during the study period, with an average of 2861 cases per year, and an overall detection rate of 20.41 cases per 100,000 inhabitants (Table 1). Socio-demographic characteristics of cases are depicted in Table 1. Crude case detection rates were significantly higher among the older age groups, as compared to <15-year-olds, and among Asian ethnic group, residents in medium-sized cities and those living outside the state capital. With the exception of the Southwest, all regions had significantly higher detection rates as compared to the Northeast region of the State (Table 1).

There was a total of 3219 (8.0%) cases in <15-year-olds, an average of 230 cases per year, with a mean case detection rate of 5.71 cases per 100,000 inhabitants. Socio-demographic characteristics of cases in children are depicted in Table 2. Case detection was significantly higher among children of all ethnicities other than Caucasian. Children living in medium-sized cities presented higher risk. With the exception of the Southwest of the State, all regions showed significantly higher detection rates as compared to the Northeast (Table 2).

The proportion of new cases with G2D was 4.80% (1921/40,054), and G2D per million people was 9.80. Details of new cases diagnosed with G2D are presented in Table 3. The detection of new cases with G2D at diagnosis was significantly higher among males, ≥70-year-olds and among Afro-Brazilians (Table 3). Residency in medium and small-sized cities and outside the state capital was associated with a higher risk of new cases with G2D. In the far south region, the G2D detection rates were highest (Table 3).

### 3.2. Spatiotemporal Analyses

**Figure 2:**
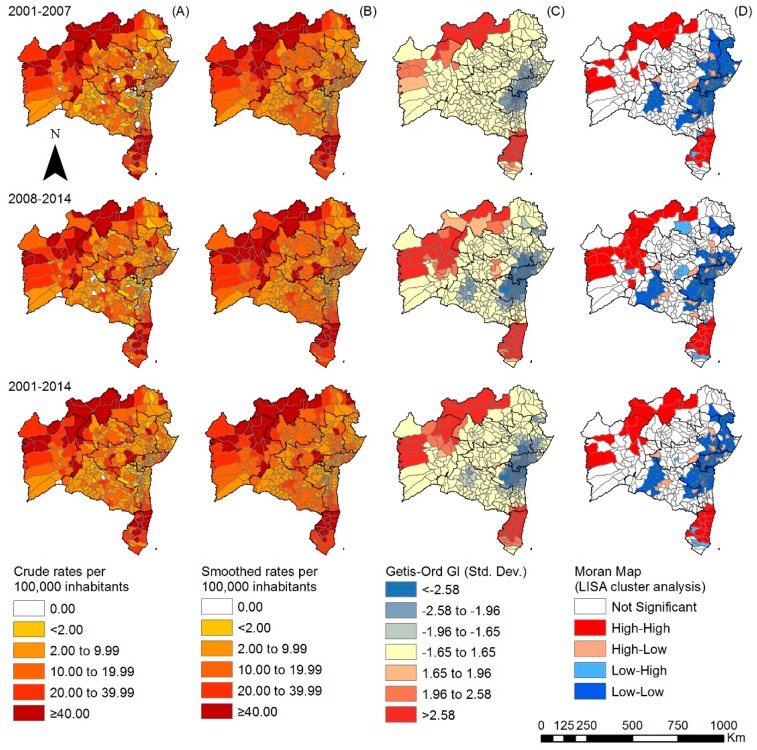
Spatiotemporal distribution of the overall new case detection rate of leprosy by municipality, Bahia state, 2001–2014: (**A**) crude detection rates (per 100,000 inhabitants); (**B**) Bayesian-smoothed detection rate (per 100,000 inhabitants); (**C**) hot-spot analysis (Getis-Ord Gi*) and (**D**) LISA cluster analysis (Moran Map).

The spatial distribution of overall crude detection rates showed that in the first observation period (2001–2007), most municipalities reported cases (93.5%, 390); 172 (44.1%) of these were classified as medium endemic, 90 (23.1%) as highly endemic and 52 (13.3%) as hyperendemic (Figure 2A). In the State’s North, West and Far South regions we identified the highest proportions of highly endemic and hyperendemic municipalities. In the following period (2008–2014), the number of municipalities with medium endemicity (48.5%, 193) increased and those with hyperendemicity (8.3%, 33) reduced. After smoothing, the distribution patterns remained similar, but spatial high-risk areas became more obvious (Figure 2B). Spatial association using Getis-Ord Gi* identified high-risk clusters for leprosy detection in the North, West and Far South regions of Bahia, over the entire observation period. Clusters of low risk were identified in the South, East and Central-East regions (Figure 2C). Spatial presentation using local Moran’s index recognized areas of spatial autocorrelation in the North, West and Far South regions (Figure 2D). These areas occurred throughout the entire observation period.

**Figure 3:**
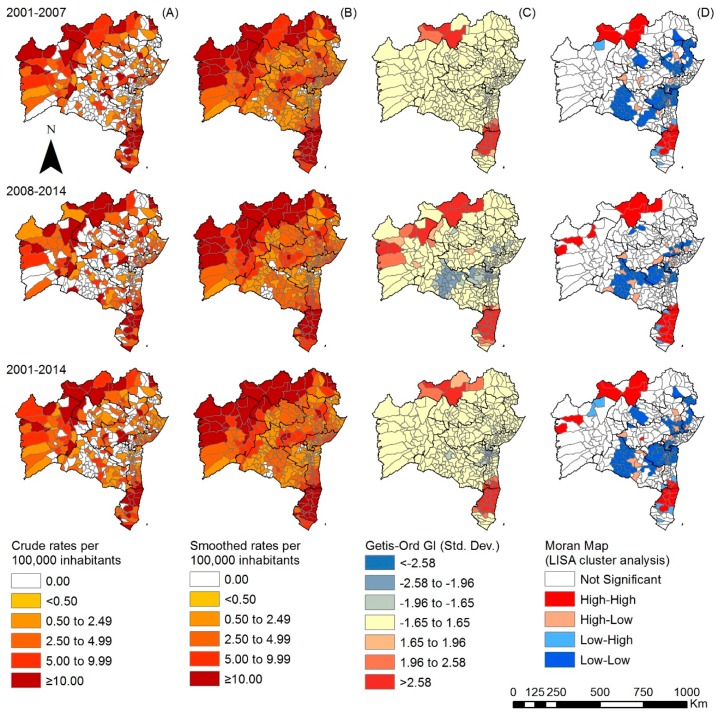
Spatiotemporal distribution of the overall new case detection rate of leprosy in <15 year-olds by municipality, Bahia state, 2001–2014: (**A**) crude detection rates of new cases of leprosy (per 100,000 inhabitants); (**B**) Bayesian-smoothed detection rate (per 100,000 inhabitants); (**C**) hot-spot analysis (Getis-Ord Gi*) and (**D**) LISA cluster analysis (Moran Map).

For the population <15 years of age, the spatial analysis of crude detection rates revealed a high number of municipalities with high endemicity (Figure 3A). In the first period analyzed (2001–2007), among the 225 (53.9%) municipalities that registered cases in this age group, 54 (28.1%) presented high endemicity. During 2008–2014, the number of highly-endemic municipalities (31.5%, 57) increased (Figure 3A). These patterns were similar after smoothing (Figure 3B). Getis-Ord Gi* analysis (Figure 3C) indicated the existence of high-risk clusters, initially in the North and Far South regions (2001–2008) which persisted during the second period (Figure 3C). A new high-risk cluster appeared in the western part of the State. The local Moran’s index confirmed high detection clusters in the previously described regions and indicated the emergence of a cluster in the Far South region with low detection (Figure 3D).

**Figure 4:**
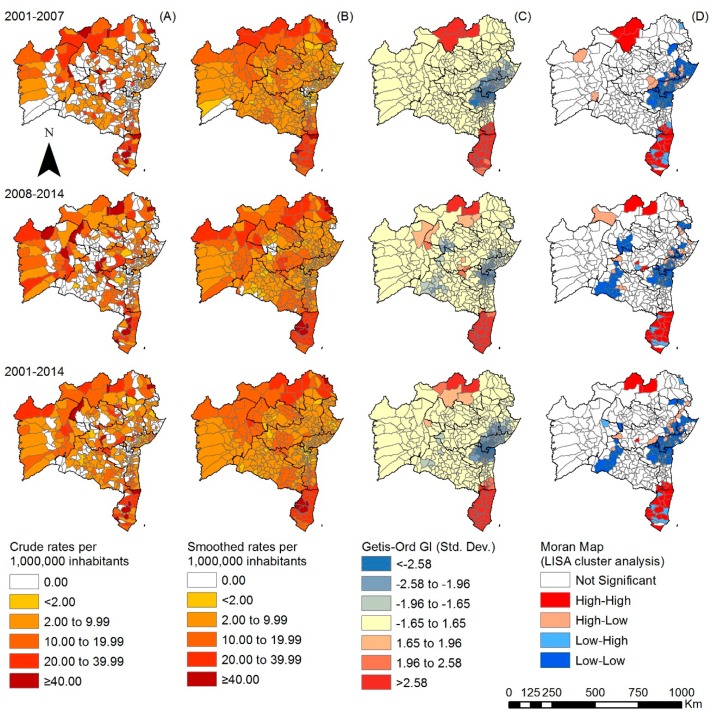
Spatiotemporal distribution of G2D per million people by municipality, Bahia state, 2001–2014: (**A**) crude detection rate of new cases of leprosy (per 1,000,000 inhabitants); (**B**) Bayesian-smoothed detection rate (per 1,000,000 inhabitants); (**C**) hot-spot analysis (Getis-Ord Gi*) and (**D**) LISA cluster analysis (Moran Map).

During the first period, new cases with G2D at the time of diagnosis were recorded in 189 (54.6%) municipalities (Figure 4A), most with less than 1 case with G2D per million people; 8.0% of all municipalities had a rate of more than 4 cases per million people, mainly in the North, West and Far South regions. The Getis-Ord Gi* analysis indicated agglomeration in the North, West and Far South regions, the latter being larger in extent (Figure 4C). During the second period, there was a general increase of the number of municipalities composing the main clusters. The local Moran’s index also indicated the existence of clusters, with high rates in the North and Far South, in addition to low rates, in a small area in the South of the State (Figure 4D).

## 4. Discussion

This is the first study assessing systematically the epidemiology and distribution of leprosy in the state of Bahia in northeast Brazil. Despite intensive control programs, the State has sustained high levels of endemicity over a period of 14 years, including a significant proportion of children and people with G2D at the time of diagnosis. Among the new cases, people aged ≥50 years were most heavily affected, with no significant gender differences. Most municipalities were defined as medium, highly endemic or hyperendemic, according to definitions of the Brazilian MoH [4].

Medium-sized cities and the extreme south of the State showed a higher leprosy risk than the other areas. Spatiotemporal patterns were heterogeneous, but in general the different indicators and analytical tools applied revealed similar high-risk areas for detection, recent transmission and late diagnosis. In fact, the occurrence of well-defined clusters was confirmed for the three Brazilian MoH/WHO indicators, using different spatial analysis techniques, mainly in the North, West and South regions of the State.

Males had a strikingly higher risk for G2D at diagnosis than females. In fact, healthcare-seeking behavior is known to be different in males, and this group usually presents less commonly and later to the health system than the female population. The reasons for this are multiple and include a historically-built way of living masculinity, which renders men negligent regarding their own health [13], a higher stigmatization, but also simply practical issues such as opening hours which often are not practical for the working population. Consequently, late diagnosis of leprosy is more common in males, reaffirming the social aspects of the disease from a gender perspective [7,18,19].

Another study conducted in Brazil from 2001 to 2013 revealed a significantly higher number of cases in men than in women, and the likelihood of presenting multibacillary cases and G2D at diagnosis was twice as high among men [20]. Another possible explanation relates to physiological risk factors [21].

However, the way that health services are organized and develop their actions can improve access to healthcare, especially prevention activities in primary health care [18,19]. A special focus should be given to the male population regarding prevention and early detection of the disease [4,6]. We suggest further the integration of specific actions of leprosy health surveillance to the National Policy of Comprehensive Attention to Men’s Health in Brazil, and to the National Policy of Workers’ Health. However, issues related to leprosy stigma should be considered for the development of these measures [22].

We observed that the risk of leprosy increased with age, which may be related to the aging population in Brazil, as well as humoral factors and possible functional impairments, which may affect social engagement, including access to health services [23]. Other studies also revealed an immunological deregulation with aging [20,24]. The changes in the immune system of the elderly may contribute to the increase of susceptibility to infectious and degenerative diseases, including leprosy [25].

The Brazilian MoH already highlighted the higher incidence, more common late diagnosis and higher risk for multibacillary disease among older age groups. Consequently, the elderly population should be a focus of specific strategies directed not only at early diagnosis, but at the systematic follow-up during and after treatment as a chronic disease, in view of the high occurrence of comorbidities, leprosy reactions, drug interactions and progression of functional limitations [19,24,25,26,27]. Specific strategies are important, such as active case finding at the residence, and measures to avoid lost opportunities for diagnosis at health centers. The Family Health Strategy should be understood as crucial in this context, as it counts on guidelines for comprehensive and longitudinal care to families, especially to those with high vulnerability [27,28].

A previous study revealed that among the elderly in Brazil the development of illnesses and disabilities causing dependence have been more frequent in the lower income strata. Therefore, in addition to expanding access to health services and actions, measures aimed at improving living conditions are necessary, reducing social inequities [23,26].

In our study, Afro-Brazilian, Asian and Amerindian ethnic groups showed a significantly higher risk for leprosy detection, and/or late diagnosis. These results indicate possible inequalities in access to health services related to ethnicity [29]. Considering the scars produced by a society where Afro-Brazilians and Amerindians historically suffered from slavery and systematic oppression, until today the social position occupied by many keeps them in a condition of higher vulnerability to diseases, especially infectious diseases and more specifically neglected tropical diseases such as leprosy, schistosomiasis, Chagas’ disease, and leishmaniasis [30]. Interventions should be made prioritizing this population, since both ethnicities are very common in the state of Bahia [12], implying a priority attention, promoting equity of care and attention in the health network. In the context of social vulnerability, the control of leprosy should be based on broad social reforms considering the social determinants of health [29], contrary to the current political and social reality of the country based on fiscal austerity measures.

The highest relative risk of occurrence of cases was observed in medium-sized cities, but more than half of the cases were registered in small cities. In the state of Bahia, in small-sized municipalities, there are usually no specialized services for leprosy available [31]. Therefore, in these areas, diagnosis and treatment also of complicated cases are strongly linked to primary health care [4]. Consequently, health professionals should be trained systematically to perform diagnosis, treatment and psychosocial rehabilitation of cases. However, several studies revealed operational difficulties of primary health services in the development of leprosy control measures [5,6,32], such as contact tracing [6]. Therefore, municipalities of medium and small size should be prioritized and must receive attention from State and Federal governments in order to strengthen regional support networks. A previous study from the state of Bahia revealed that from 2003 to 2014, only 47% of leprosy contacts were examined [5]. The proportion of cases diagnosed by contact tracing reduced from 18% in 2004 to 8% in 2014. It is important to focus on contact surveillance as a priority strategy for the control of leprosy, following the recommendation of the Brazilian Ministry of Health [1,4,5]. More than 80 municipalities of Bahia maintained high or very high detection rates in children, evidencing considerable ongoing transmission [4]. There are clear difficulties for timely diagnosis also in this age group. Diagnosis often requires the performance of specialists, given its clinical complexity especially in children. Similar scenarios have been identified in municipalities in other states and regions of Brazil [10]. A study carried out in the urban area of the capital city of Salvador, Bahia, recognized an increased risk of occurrence in children <15 years in 22 neighborhoods, with an average occurrence of at least 10 cases per 100,000 inhabitants [33]. Another longitudinal study in the state of Bahia revealed that although the general detection showed a tendency of reduction, in children <15 years, there was maintenance of detection rates for more than a decade [34].

The WHO global strategy for the period 2016–2020 defined one of the main targets as the reduction in the number of children diagnosed with leprosy, and zero visible deformities [1]. A joint effort of health services, universities, and social movements is required in order to provide quality access for children [35].

The spatial analyses of new cases with G2D revealed late diagnosis in the great majority of the municipalities of Bahia, enhancing transmission [1,4]. A previous study from the State evidenced a significant increase of people with G2D [35] over time. An ecological study involving the states of Mato Grosso, Tocantins, Pará, Maranhão and Rondônia, between 2001 and 2012, recognized that the rate of new cases with G2D was stable, varying from 3.62 cases per 100,000 inhabitants in 2001 to 3.41 cases per 100,000 inhabitants in 2012. [36]. The physical disabilities caused by leprosy pervert the conditions of poverty and leprosy-related stigma [22]. In addition to efficient rehabilitation services, it is necessary to establish community-based rehabilitation strategies, promote social inclusion, empower the population and enhance social participation [37]. These strategies are fundamental to break the cycles of vulnerability, demanding intersectoral and sustainable measures [9].

Local autocorrelation methods emphasized the existence of clusters with statistical significance among the epidemiological indicators analyzed. The clusters indicate an increased risk of transmission, active circulation of *M. leprae* and a high number of cases with advanced disability. The results of the Local and Getis-Ord Gi* Moran parameters confirmed two significant clusters in the north and the south. Other studies of this nature revealed several municipality clusters for high leprosy transmission and late diagnosis in an endemic area using different statistical approaches [38].

Intensive monitoring of high risk areas is crucial in order to institute more comprehensive surveillance measures, and to provide comprehensive longitudinal care during and after specific treatment, including social rehabilitation measures and stigma coping [22,37]. In addition, the detection of under-notifications and hidden endemic scenarios in neighboring areas is important, as low endemicity levels in some areas may be related to the poor quality of health services in the realm of active case finding and early diagnosis, not due to the absence of transmission [5,6,32].

For sustainable control, the historical, social, economic and cultural contexts of endemic areas must be considered in an integrative manner [29]. It is necessary to carry out future studies, focusing on individual, social, and programmatic dimensions of vulnerability. The maintenance of high levels of leprosy endemicity reaffirms the need for interdisciplinary research and for constructing dynamic prevention measures and health promotion [39].

The study presents limitations regarding the use of secondary databases, considering non-completeness and inconsistencies for some variables. However, the incorporation of the state database in a historical series of 14 years, together with the need for studies with this approach in the state of Bahia, justifies its use. The definition of the clusters did not allow to delimit their borders with high accuracy, even with the high probability of their existence. A low-frequency area surrounded by areas with a higher number of cases was included in a cluster, although it may have different characteristics. The integration of different analytical techniques has increased consistency of the evidence provided in this study. Considering the large number of municipalities in the State with a small population, the incorporation of the local spatial smoothing method to the analysis was a useful tool for monitoring and surveillance of leprosy. This occurs not only because it is a rare event in some municipalities, but because they often have a small population. The analysis is thus a practical approach to estimate underreported cases, a common reality in different municipalities of Brazil [40].

We conclude that leprosy persists as a relevant public health problem in the state of Bahia. The identified spatiotemporal patterns revealed the maintenance of high-risk clusters for detection, transmission and late diagnosis. We reinforce the importance of integrating surveillance, prevention and control actions in regions of higher risk of leprosy detection and disabilities, and in the most vulnerable populations.

## Figures and Tables

**Table 1:** Sociodemographic characteristics of leprosy cases and associated factors in Bahia state, 2001–2014.

Variables	Cases (Total)	Average Cases Annually (2001–2014) n (%)	Detection Rate ^a^	95% CI	RR	95% CI	*p* Value
Gender ^b^							
Male	20,132	1438 (50.3)	20.75	19.70–21.85	1.03	0.96–1.11	0.3900
Female	19,922	1423 (49.7)	20.08	19.07–21.15	Ref	-	-
Age group (years) ^b^							
<15	3219	230 (8.0)	5.44	4.78–6.19	Ref	-	-
15–29	9330	666 (23.3)	15.87	14.70–17.12	2.91	2.51–3.39	<0.0001
30–39	7092	507 (17.7)	26.15	23.99–28.55	4.81	4.11–5.62	<0.0001
40–49	6706	479 (16.7)	32.85	30.04–35.93	6.03	5.16–7.06	<0.0001
50–59	6052	432 (15.1)	43.86	39.89–48.16	8.05	6.86–9.45	<0.0001
60–69	4015	287 (10.0)	44.30	39.49–49.76	8.14	6.85–9.68	<0.0001
≥70	3640	260 (9.1)	46.03	40.76–51.97	8.45	7.08–10.09	<0.0001
Ethnicity ^b^							
Caucasian	6914	494 (20.1)	16.10	14.74–17.58	Ref	-	-
Afro-Brazilian	6188	442 (18.0)	22.22	20.24–24.39	1.38	1.33–1.43	<0.0001
Asian	345	25 (1.0)	26.97	18.50–40.40	1.68	1.50–1.87	<0.0001
Mixed/Pardo-Brazilian	20,799	1486 (60.5)	19.15	18.20–20.20	1.19	1.16–1.22	<0.0001
Amerindian	156	11 (0.5)	18.48	10.20–32.70	1.15	1.00–1.35	0.0881
City size (by inhabitants)							
Small (<100,000)	21,348	1525 (53.3)	18.10	17.21–19.03	1.34	1.21–1.49	<0.0001
Medium (100,000–500,000)	12,279	877 (30.7)	37.66	35.25–40.23	2.80	2.50–3.13	<0.0001
Large (>500,000)	6433	460 (16.1)	13.45	12.29–14.75	Ref	-	-
Residing in the state capital							
Yes	4962	354 (12.4)	12.96	11.66–14.36	Ref	-	-
No	35,098	2507 (87.6)	22.22	21.37–23.11	1.72	1.54–1.92	<0.0001
Region ^b^							
North	7916	565 (19.8)	55.65	51.21–60.38	6.18	4.85–7.88	<0.0001
Northeast	1030	74 (2.6)	8.93	7.16–11.28	Ref	-	-
South	2791	199 (7.0)	11.87	10.31–13.62	1.32	1.01–1.72	0.0422
South-west	2635	188 (6.6)	10.76	9.32–12.40	1.20	0.91–1.57	0.1912
East	7677	548 (19.2)	12.27	11.28–13.33	1.36	1.07–1.74	0.0121
Central East	4457	318 (11.1)	15.37	13.76–17.13	1.71	1.33–2.20	<0.0001
West	5576	398 (13.9)	47.67	43.18–52.55	5.30	4.13–6.79	<0.0001
Far south	6252	447 (15.6)	58.91	53.75–64.69	6.56	5.13–8.39	<0.0001
Central North	1725	123 (4.3)	15.98	13.40–19.00	1.78	1.33–2.37	0.0001
Total	40,060	2861 (100.0)	20.41	19.68–21.17	-	-	-

CI: confidence intervals; RR: relative risk; -: not calculated; ^a^ The average annual detection rate (per 100,000 inhabitants), based on the calculation of the average number of new cases in the period of fourteen years as the numerator and the size of the population in the middle of the study period, as the denominator. Population data on ethnicity were obtained from the Brazilian national census (2000 and 2010). The number of the population in relation to ethnicity, for the middle of the period, was derived from the Continuous National Household Sample Survey (PNAD) estimates; ^b^ Data not available in all cases (Gender: 6 cases, Age group: 6 cases, Ethnicity: 5658 cases, Health Regions: 1 case).

**Table 2:** Sociodemographic characteristics of leprosy cases in <15 year-olds and associated factors in Bahia state, 2001–2014.

Variables	Cases	Average Annual (2001–2014) n (%)	Detection Rate in Children ^a^	95% CI	RR	95% CI	*p* Value
Gender							
Male	1585	113 (49.2)	5.51	4.59–6.63	Ref	-	-
Female	1634	117 (50.8)	5.90	4.92–7.07	1.35	1.22–1.50	<0.0001
Ethnicity ^b^							
Caucasian	448	32 (16.4)	3.51	2.49–4.95	Ref	-	-
Afro-Brazilian	475	34 (17.4)	7.25	5.19–10.12	2.06	1.81–2.35	<0.0001
Asian	25	2 (0.9)	8.05	2.21–29.34	2.05	1.37–3.06	0.0005
Mixed/Pardo-Brazilian	1767	126 (64.6)	5.15	4.33–6.14	1.47	1.33–1.63	<0.0001
Amerindian	21	2 (0.8)	12.38	3.40–45.13	2.65	1.71–4.10	<0.0001
City size (inhabitants)							
Small (<100,000)	1735	124 (53.9)	4.89	4.10–5.83	1.76	1.36–2.28	<0.0001
Medium (100,000–500,000)	970	69 (30.1)	10.42	8.24–13.19	4.70	3.65–6.06	<0.0001
Large (>500,000)	514	37 (16.0)	4.43	3.22–6.11	9.85	7.71–12.59	<0.0001
Residing in the state capital							
Yes	422	30 (13.1)	4.39	3.08–6.27	Ref	-	-
No	2797	200 (86.9)	5.97	5.20-6.86	1.35	1.22–1.50	<0.0001
Region							
North	731	52 (22.7)	16.70	12.74–21.89	8.25	6.47–10.53	<0.0001
Northeast	71	5 (2.2)	2.00	0.86–4.69	Ref	-	-
South	197	14 (6.1)	2.84	1.69–4.77	1.40	1.07–1.84	0.0141
South-west	120	9 (3.7)	1.85	0.97–3.52	0.87	0.65–1.16	0.3442
East	683	49 (21.2)	4.30	3.25–5.68	2.11	1.65–2.69	<0.0001
Central East	308	22 (9.6)	3.58	2.36–5.41	1.76	1.36–2.28	<0.0001
West	362	26 (11.2)	9.60	6.56–14.07	4.70	3.65–6.06	<0.0001
Far south	659	47 (20.5)	19.99	15.03–26.58	9.85	7.72–12.59	<0.0001
Central North	88	6 (2.7)	2.59	1.19–5.65	1.33	0.98–1.82	0.0706
Total	3219	230 (100.0)	5.71	5.02–6.49	-	-	-

CI: confidence intervals; RR: relative risk; -: not calculated; ^a^ Average annual detection rate (per 100,000 inhabitants), based on the calculation of the average number of new cases in the period of fourteen years as the numerator and the size of the population in the middle of the study period, as the denominator. Population data on ethnicity were obtained from the Brazilian national census (2000 and 2010). The number of the population in relation to ethnicity, for the middle of the period, was derived from PNAD estimates; ^b^ Data not available in all cases (Ethnicity: n = 483).

**Table 3:** Sociodemographic characteristics of leprosy cases with G2D at diagnosis and factors in Bahia state, 2001–2014.

Variables	Cases	Average Annual (2001–2014) n (%)	Detection Rate ^a^	95% CI	RR	95% CI	*p* Value
Gender							
Male	1351	97 (70.3)	14.00	11.48–17.08	2.43	2.20–2.67	<0.0001
Female	570	41 (29.7)	5.80	4.28–7.87	Ref	-	-
Age group (years)							
<15	56	4 (2.9)	0.98	0.38–2.52	Ref	-	-
15–29	300	21 (15.6)	5.00	3.27–7.64	5.39	4.05–7.17	<0.0001
30–39	310	22 (16.1)	11.40	7.53–17.26	12.07	9.08–16.05	<0.0001
40–49	316	23 (16.4)	15.80	10.53–23.71	16.35	12.31–21.73	<0.0001
50–59	323	23 (16.8)	23.30	15.53–34.96	24.73	18.62–32.84	<0.0001
60–69	286	20 (14.9)	30.90	20.00–47.73	33.33	25.03–44.39	<0.0001
≥70	330	24 (17.2)	42.50	28.56–63.24	44.08	33.21–58.51	<0.0001
Ethnicity ^b^							
Caucasian	342	24 (20.0)	7.80	5.24–11.61	Ref	-	-
Afro-Brazilian	353	25 (20.7)	12.60	8.54–18.60	1.59	1.37–1.85	<0.0001
Asian	15	1 (0.9)	10.90	1.92–61.74	1.47	0.88–2.47	0.1420
Mixed/Pardo-Brazilian	986	70 (57.7)	9.00	7.12–11.37	1.14	1.00–1.29	0.0364
Amerindian	13	1 (0.8)	16.60	2.93–94.02	1.93	1.11–3.37	0.0196
City size (inhabitants)							
Small (<100,000)	974	70 (50.7)	8.30	6.57–10.49	1.16	1.02–1.31	0.0222
Medium (100,000–500,000)	605	43 (31.5)	18.50	13.74–24.92	2.60	2.27–2.96	<0.0001
Large (>500,000)	342	24 (17.8)	7.00	4.70–10.42	Ref	-	-
Residing in the state capital							
Yes	254	18 (13.2)	6.60	4.18–10.43	Ref	-	-
No	1667	119 (86.8)	10.50	8.78–12.56	1.59	1.39–1.82	<0.0001
Region							
North	322	23 (16.8)	22.60	15.06–32.91	5.02	3.74–6.73	<0.0001
Northeast	52	4 (2.7)	4.90	1.91–12.60	Ref	-	-
South	174	12 (9.1)	7.10	4.06–12.41	1.64	1.20–2.24	0.0017
South-west	187	13 (9.7)	7.40	4.33–12.66	1.69	1.25–2.30	0.0008
East	387	28 (20.1)	6.30	4.36–9.11	1.37	1.03–1.83	0.0324
Central East	220	16 (11.5)	7.70	4.74–12.51	1.68	1.24–2.28	0.0007
West	121	9 (6.3)	10.80	5.68–20.53	2.29	1.66–3.17	<0.0001
Far south	357	26 (18.6)	34.30	23.41–50.26	7.46	5.58–9.98	<0.0001
Central North	101	7 (5.3)	9.10	4.41–18.78	2.07	1.49–2.90	<0.0001
Total	1921	137 (100.0)	9.80	8.29–11.58	-	-	-

CI: confidence intervals; RR: relative risk; -: not calculated; ^a^ The average annual detection rate (per million people), based on the calculation of the average number of new cases in the period of fourteen years as the numerator and the size of the population in the middle of the study period, as the denominator. Population data on ethnicity were obtained from the Brazilian national census (2000 and 2010). The number of the population in relation to ethnicity, for the middle of the period, was derived from PNAD estimates; ^b^ Data not available in all cases (Ethnicity: n = 212).
